# Plasma proteomics identifies four candidate biomarkers associated with macrovascular complications in type 2 diabetes: evidence from a clinical cohort and an animal model

**DOI:** 10.3389/fendo.2026.1955597

**Published:** 2026-10-30

**Authors:** Nuo Chen, Jingxuan Wang, Xiaojing Yuan, Wan Zhou

**Affiliations:** 1 Department of Endocrinology and Metabolism, The First Affiliated Hospital of USTC, Division of Life Sciences and Medicine, University of Science and Technology of China, Hefei, China; 2 Division of Life Sciences and Medicine, University of Science and Technology of China, Hefei, China

**Keywords:** ADSL, biomarkers, LGALS3, macrovascular disease, MGAT1, proteomics, type 2 diabetes, VSIG4

## Abstract

**Objective:**

To identify and validate plasma protein biomarkers for T2DM macrovascular complications through proteomic screening, clinical validation, and animal models.

**Methods:**

Plasma proteomics (4D-DIA, n = 6/group) was used to screen candidate proteins via differential expression, WGCNA, and PPI analyses. Candidates were validated in an independent clinical cohort (n = 162: 41 healthy controls, 35 T2DM alone, 86 T2DM with macrovascular complications) by ELISA for associations with metabolic parameters and diagnostic performance. A diabetic atherosclerosis mouse model (n = 10/group; C57BL/6J for control and diabetic groups, ApoE−/− for diabetic atherosclerosis, 24 weeks) was used to assess protein/mRNA expression and atherosclerotic lesions by Western blot, RT-qPCR, histology, and immunohistochemistry, with correlations to inflammatory and metabolic markers.

**Results:**

In an exploratory proteomic discovery phase using a very small cohort (n = 6 per group), four candidate proteins were prioritized: adenylosuccinate lyase (ADSL), galectin-3 (LGALS3), mannosyl (α-1,3-)-glycoprotein β-1,2-N-acetylglucosaminyltransferase (MGAT1), and V-set and immunoglobulin domain-containing protein 4 (VSIG4). The four-protein panel showed good discrimination (AUC = 0.961). Adding the panel to a clinical model (age, disease duration, and HbA1c) significantly improved diagnostic performance (ΔAUC = 0.138, P = 0.008; NRI = 0.314; IDI = 0.261), with good calibration and internal stability. Firth penalized logistic regression identified ADSL (OR = 0.327, P = 0.0199) and MGAT1 (OR = 7.435, P = 2.71×10^–6^) as independently associated with prevalent macrovascular disease. In animal models, ADSL, LGALS3, and VSIG4 correlated significantly with seven indicators, while MGAT1 correlated significantly with LDL-C, HDL-C, IL-6, and TNF-α after FDR. LGALS3 and VSIG4, though not independent in multivariable analysis, showed consistent upregulation and moderate discriminative ability (AUC = 0.736 and 0.686), supporting biological relevance.

**Conclusion:**

ADSL and MGAT1 were independently associated with prevalent macrovascular complications.LGALS3 and VSIG4 may reflect cumulative metabolic and inflammatory burden rather than a lack of biological relevance; animal studies provide supportive evidence for this hypothesis.The four-protein panel improved diagnostic discrimination over clinical variables alone. These findings are exploratory and based on a small discovery cohort and internal validation; the four proteins are therefore best regarded as candidate diagnostic biomarkers associated with prevalent disease. Prospective external validation is warranted.

## Introduction

Type 2 diabetes mellitus (T2DM) is a chronic metabolic disease primarily characterized by insulin resistance and progressive decline in pancreatic β-cell function (1). In recent years, with the accelerating aging of the population, the Westernization of lifestyles, and the continued rise in obesity rates, T2DM has become one of the chronic diseases with the fastest-growing global disease burden (2). According to data from the International Diabetes Federation (IDF), the global prevalence of diabetes among adults aged 20–79 reached 10.5% in 2021 (approximately 536.6 million people) and is projected to rise to 12.2% by 2045 (approximately 783.2 million people), with China having the largest number of people with diabetes worldwide (3). In addition to the metabolic abnormalities caused by hyperglycemia itself, various chronic complications — particularly cardiovascular and cerebrovascular complications — have become major factors affecting patients’ quality of life and long-term prognosis (2).

Macrovascular complications of T2DM primarily manifest as coronary atherosclerotic heart disease, stroke, and peripheral artery disease, with accelerated and diffuse atherosclerosis serving as their pathological basis (4). Studies have shown that prolonged exposure to a high-sugar environment can promote vascular damage through various mechanisms, including the induction of oxidative stress, the release of inflammatory factors, the accumulation of advanced glycation end products (AGEs), and endothelial dysfunction (5). At the same time, processes such as lipid metabolism disorders, immune-metabolic imbalances, and abnormal proliferation and migration of vascular smooth muscle cells collectively contribute to the formation and progression of atherosclerotic plaques (6). However, the pathogenesis of macrovascular complications in T2DM is complex, involving the coordinated regulation of multiple signaling pathways and various cell types, and has not yet been fully elucidated.

Although imaging tests and risk factor assessment systems have been continuously refined in recent years, the identification of macrovascular complications in T2DM remains a significant challenge. Traditional biomarkers, such as lipid profiles, inflammatory factors, and markers of endothelial damage, have certain limitations in both sensitivity and specificity, making it difficult to meet the demands for diagnostic stratification and individualized management. Therefore, identifying novel biomarkers with high diagnostic performance and potential for clinical translation has become a key focus in research on diabetic complications.

Proteomics technologies enable systematic and holistic analysis of disease-associated protein expression profiles, providing powerful tools for elucidating disease mechanisms and identifying potential biomarkers (7). Among these, DIA quantitative proteomics offers high throughput, high sensitivity, and good reproducibility, and has been widely applied in biomarker screening for various diseases. Furthermore, bioinformatics methods such as WGCNA and PPI network analysis can identify key regulatory proteins from complex data, improving the reliability and accuracy of candidate biomarker screening.

Current research on proteins associated with macrovascular complications of T2DM has largely focused on the validation of individual proteins or the exploration of localized mechanisms, lacking a systematic research approach that spans from omics-based discovery to clinical validation and animal model verification. Therefore, this study employs a comprehensive research strategy comprising proteomic screening, validation in an independent clinical cohort, and animal model verification to systematically screen and validate key proteins closely associated with T2DM macrovascular disease. The aim is to identify candidate proteins associated with prevalent macrovascular complications in T2DM and to evaluate their diagnostic discrimination, rather than to predict future cardiovascular events or establish causality.

## Materials and methods

### Study design

This study employs a research design involving discovery cohort screening, clinical independent cohort validation, and animal model verification to systematically screen and validate biomarkers associated with macrovascular complications of T2DM. The study process includes:

1. Proteomics Discovery Phase: Using 4D-DIA (diaPASEF) quantitative proteomics technology, we performed high-throughput analysis on plasma samples from the normal control group (Pro-NC), the type 2 diabetes-only group (Pro-DM), and the diabetes with macrovascular complications group (Pro-DA). Key candidate proteins were identified through differential expression analysis, WGCNA, and protein-protein interaction (PPI) network analysis.
2. Clinical Validation Phase: An independent clinical validation cohort was established, and ELISA was used to measure the expression levels of candidate proteins and analyze their correlation with clinical indicators and diagnostic performance.
3. Animal Validation Phase: A mouse model of diabetic atherosclerosis was established to provide supportive evidence for the expression patterns of candidate proteins during disease onset and progression. Supportive analyses were conducted at three levels: histopathology, protein expression, and gene transcription. Subsequently, the correlations between target proteins and biochemical and inflammatory markers were further investigated. Because the Mod-DA group used an ApoE−/− background while the other groups used C57BL/6J mice, group differences may reflect diabetes, atherosclerosis, and genetic background effects combined. Animal data were therefore considered supportive.

### Proteomics discovery phase

#### Study population

A total of 18 subjects were recruited for the discovery phase and divided into three groups (n = 6 per group): healthy controls (Pro-NC), type 2 diabetes mellitus alone (Pro-DM), and T2DM with macrovascular complications (Pro-DA). The Anhui Provincial Hospital Ethics Committee approved the protocol (No. 2023KY402); all subjects gave written informed consent. The study followed the Declaration of Helsinki principles.

1. Pro-NC subjects had normal fasting glucose (<6.1 mmol/L) and HbA1c (<5.7%), no diabetes history, clean vascular imaging, normal liver and kidney function, and no recent infection, surgery, or major stress.
2. The diagnostic criteria for Pro-DM followed the WHO (1999) and ADA (2020) guidelines: fasting plasma glucose (FPG) ≥ 7.0 mmol/L or 2-hour plasma glucose ≥ 11.1 mmol/L during an oral glucose tolerance test (OGTT) or glycated hemoglobin (HbA1c) ≥ 6.5%. All acute and chronic diabetic complications were excluded, including diabetic nephropathy, diabetic retinopathy, diabetic peripheral neuropathy, and various macrovascular complications.
3. Pro-DA subjects met T2DM criteria and carried documented macrovascular disease: prior MI, PCI, or CABG; coronary stenosis ≥ 50% on CTA or angiography; ischemic stroke or TIA; ABI < 0.90 or lower-extremity stenosis ≥ 50%; or carotid plaque ≥ 1.5 mm or stenosis ≥ 50%.

Exclusion criteria included type 1 diabetes, acute infection, malignancy, autoimmune disease, severe organ failure, recent major surgery, glucocorticoid or immunosuppressant use, and hemolyzed or lipemic samples.

#### Protein extraction and DIA acquisition

Plasma proteins were extracted and digested using a one-step method with 1% SDC, 100 mM Tris-HCl (pH 8.5), 10 mM TCEP, and 40 mM CAA at 60°C for 1 h. Trypsin was added at a 1:50 enzyme-to-protein ratio and incubated overnight at 37°C. Peptides were desalted using SDB columns. LC-MS/MS analysis was performed on a timsTOF Pro mass spectrometer coupled with an UltiMate 3000 RSLCnano system using a 60-min gradient, operated in diaPASEF (DIA) mode. Raw DIA data were processed using DIA-NN (v1.8) against the UniProt Human proteome database. A predicted spectral library and the MBR function were used for protein identification and quantification. Results were filtered at a 1% FDR at both precursor and protein levels.

#### Analysis of differentially expressed proteins

Using R (v4.3.0) and limma, we standardized the abundance matrix, corrected for batch effects, and fit a linear model (design = ~0 + group). Pairwise comparisons — Pro-NC vs. Pro-DM, Pro-NC vs. Pro-DA, Pro-DM vs. Pro-DA — were tested by empirical Bayes with Benjamini-Hochberg correction. Proteins with |log_2_FC| ≥ log_2_(1.2) and FDR < 0.05 qualified as differentially abundant proteins (DAPs).

#### WGCNA

We built a protein co-expression network with the WGCNA package. The soft threshold β was selected for scale-free topology, adjacency and topological overlap matrices were computed, and modules were identified by dynamic tree cutting and merged where appropriate. Proteins with |module membership| > 0.6 and |phenotype significance| > 0.4 were flagged as candidate hubs, then intersected with DAPs to obtain core candidates. Given the limited sample size (n = 6 per group), WGCNA was performed only as an exploratory, hypothesis-generating analysis. No formal sensitivity or stability analysis—including bootstrap resampling, leave-one-out validation, module preservation analysis, or consensus WGCNA—was performed, because with six subjects per group these approaches would be severely underpowered and could produce misleadingly unstable or overfitted results. Therefore, module assignments, module–trait correlations, and hub protein identification should be considered preliminary and require confirmation in larger independent datasets.

#### Functional enrichment analysis

GO and KEGG enrichment analyses were performed using the clusterProfiler package. The hypergeometric test was employed with Benjamini-Hochberg correction for multiple comparisons, and statistical significance was defined as a false discovery rate (FDR) of q < 0.05. For gene set enrichment analysis (GSEA), 1,000 permutations were applied.

#### PPI network analysis

Candidate proteins were uploaded to STRING v11.0 with a confidence score threshold of 0.4 (medium confidence), and only experimentally validated and database-curated interactions were included. The interaction data were imported into Cytoscape v3.8.0. Isolated nodes were removed and topological parameters were calculated using the NetworkAnalyzer plugin.Hub proteins identified by MCC and DMNC were regarded as candidate proteins for downstream validation.Only proteins ranked in the top 10 by both algorithms were retained. Both ranks are reported to ensure transparency in the selection process. The final candidate list was subsequently validated in an independent cohort.

### Clinical validation phase

#### Clinical data collection

An independent clinical validation cohort was established from subjects recruited between March 2025 and March 2026. The healthy control group (Pat-NC, n = 41) comprised participants from the Health Examination Center. The diabetes group (Pat-DM, n = 35) and the diabetes with atherosclerosis group (Pat-DA, n = 86) were recruited from the Inpatient Department. The core disease definitions for the three groups in the validation cohort were consistent with those in the discovery cohort; however, additional data on blood pressure, blood glucose, blood lipids, renal function, urine albumin-to-creatinine ratio(UACR), and major medications were recorded, and relevant confounding factors were adjusted for in the multivariate analysis. The sample size was calculated based on the formula for diagnostic test precision: n = (Z<sub>)^2^ × Se × (1 − Se)/d^2^, where Se is the expected sensitivity or specificity, d is the allowable precision, and Z<sub> = 1.96 for a 95% confidence level. With an expected sensitivity of 85% and a precision of ±8%, the required number of cases was approximately 77; with an expected specificity of 80% and a precision of ±13%, the required number of controls was approximately 35. Our final sample of 162 subjects (86 cases, 35 T2DM controls, and 41 healthy controls) exceeded these requirements.The inclusion and exclusion criteria, as well as the core disease definitions for Pat-NC, Pat-DM, and Pat-DA in the validation cohort, were consistent with those in the discovery cohort. Given the high clinical heterogeneity of the validation cohort, additional data on disease duration, blood glucose levels, blood lipid levels, renal function, UACR were collected and considered as potential confounders.Major medication categories (including metformin, sulfonylureas, insulin, statins, antihypertensive agents, and antiplatelet drugs) were also recorded. However, the final multivariable model was pre-specified to include only age, disease duration, and HbA1c as clinical covariates, as described in the Statistical Analysis section.The study protocol was approved by the hospital’s ethics committee, and all participants signed written informed consent forms.

General data, including age, sex, duration of illness, body mass index (BMI), systolic blood pressure, and diastolic blood pressure, were collected from patients’ medical records. All study participants fasted for at least 8 hours overnight, and blood samples were drawn intravenously early the following morning to measure fasting blood glucose (FBG), glycated hemoglobin (HbA1c), triglycerides (TG), total cholesterol (TC), low-density lipoprotein cholesterol (LDL-C), and high-density lipoprotein cholesterol (HDL-C), as well as liver and kidney function. The Triglyceride-Glucose Index (TyG), calculated as TyG = ln((FBG × TG)/2) with both FBG and TG expressed in mg/dL, is a reliable alternative marker for assessing insulin resistance that correlates well with HOMA-IR and the hyperinsulinemic-euglycemic clamp technique without requiring insulin or C-peptide data (8). A midstream urine sample was collected the following morning to measure urinary albumin and creatinine levels and calculate the UACR for the assessment of diabetic nephropathy. The participants’ past and current medication history was recorded, including antidiabetic drugs (metformin, sulfonylureas, insulin, DPP-4 inhibitors, SGLT2 inhibitors), antihypertensive drugs (ACE inhibitors/ARBs, calcium channel blockers, diuretics), lipid-lowering drugs (statins, fibrates), and antiplatelet agents. Screening for diabetes-related complications was also performed, and data on macrovascular complications such as coronary heart disease, ischemic stroke, and peripheral artery disease were collected, including carotid ultrasound, electrocardiogram, echocardiogram, color Doppler ultrasound of the lower extremities, coronary CTA/coronary angiography, cranial CT/MRI, and records of previous confirmed diagnoses.

#### ELISA assay

Peripheral venous blood was collected from the subjects, centrifuged at 3000 rpm for 15 minutes to separate the serum, and then aliquoted and stored at −80 °C for later use. Commercial ELISA kits from Shanghai Weiao Biotechnology (EH11414M, EH10697M) and Shanghai Enzyme-Linked Biotechnology (ml058116M, ml098748M) were used to detect the expression levels of ADSL, LGALS3, MGAT1, and VSIG4 proteins in serum, following the manufacturer’s instructions. For each batch of experiments, a standard curve and quality control samples were established. Absorbance values were measured at the specific wavelengths indicated in the instructions, and protein concentrations were calculated based on the standard curve.

#### Statistical analysis

Statistical analyses were performed using R 4.3.0 and SPSS 26.0. Continuous variables were compared using ANOVA or Kruskal-Wallis tests as appropriate, and categorical variables using χ^2^ tests. Spearman’s correlation was used to assess associations between proteins and clinical parameters.

Logistic regression models were constructed for: (1) a clinical model (age + disease duration + HbA1c); (2) a four-protein model; and (3) a combined model incorporating both. Proteins were standardized to per-1-SD increments. Due to quasi-complete separation, Firth penalized logistic regression was applied (9), and P values were derived from the penalized likelihood ratio test.

The base clinical model was pre-specified to include age, disease duration, and HbA1c, as these are the most established and universally available core risk factors for macrovascular complications in T2DM. No stepwise or data-driven variable selection was performed, and all four proteins were entered simultaneously. Given the sample size and event count, adding all available variables would have reduced the events-per-variable ratio below the conventional threshold and increased the risk of overfitting; we therefore retained this parsimonious model as the primary clinical comparator. Multicollinearity was assessed using variance inflation factors (VIFs) and Spearman correlation coefficients. All VIFs were below 2, and the maximum pairwise correlation was 0.57 (age vs. disease duration), indicating no substantial multicollinearity. The complete model, including all covariates, is reported in **Supplementary Table S2**.

ROC curves were generated and AUCs calculated for each model. AUCs were compared using the DeLong test (10), and 95% CIs for ΔAUC were estimated via 1,000 bootstrap resamples. To evaluate incremental value, we calculated the categorical NRI and IDI, with risk categories defined as <20%, 20–50%, and >50% (11), which correspond to commonly used low-, intermediate-, and high-risk categories in clinical stratification.Because this is a cross-sectional diagnostic study, these thresholds are not prospectively validated, and NRI and IDI should be interpreted as exploratory measures of incremental diagnostic value.Calibration was assessed using calibration plots, calibration slope, and Brier score for the combined model. Decision curve analysis was performed to evaluate net clinical benefit (12).

Internal validity was assessed using five-fold stratified cross-validation, repeated 50 times, for both the clinical and combined models. Optimism was calculated as apparent AUC minus CV-AUC. All tests were two-sided with P < 0.05 considered statistically significant.Model construction and performance evaluation were performed in the same clinical validation cohort. Therefore, the reported performance represents internal validation. Five-fold stratified cross-validation was used to assess internal stability, but it does not replace external validation in an independent cohort.

For correlation analyses involving multiple comparisons, Benjamini-Hochberg FDR correction was applied, and q < 0.05 was considered statistically significant.

### Animal validation phase

#### Laboratory animals and model development

SPF-grade male C57BL/6J mice and ApoE−/− mice aged 6–8 weeks were selected. All animals were housed in a standard containment facility at a temperature of 22 ± 2 °C and a humidity of 55 ± 5%, with a 12-hour light-dark cycle and free access to food and water. The experimental protocol was approved by the Animal Ethics Committee. After a 1-week acclimatization period, the animals were divided into groups using the random number table method. The Mod-NC group (n = 10, C57BL/6J) was fed a standard diet; the Mod-DM group (n = 10, C57BL/6J) was fed a high-fat diet combined with STZ to induce a diabetic model; and the Mod-DA group (n = 10, ApoE−/−) was fed a high-fat diet combined with STZ to induce a diabetic atherosclerosis model. To ensure plaque formation in the Mod-DA group, all mice were maintained on their respective diets for 24 weeks. By the end of the study, one mouse in the Mod-DM group and two mice in the Mod-DA group had died; all surviving mice had successfully developed the disease model.

Diabetes Model Establishment: Beginning in the fifth week, STZ was dissolved in pre-chilled 0.1 mol/L citric acid-sodium citrate buffer (pH 4.5). The solution was stored refrigerated and protected from light, and injections were completed within 20 minutes. Mice in the Mod-DM and Mod-DA groups received daily intraperitoneal injections of STZ (40 mg/kg) for 5 consecutive days, while those in the Mod-NC group received intraperitoneal injections of an equal volume of citrate buffer during the same period. One week later, tail vein blood glucose levels were monitored continuously. Diabetes onset was defined as a random blood glucose level of ≥ 11.1 mmol/L accompanied by classic symptoms of hyperglycemia (polyuria, polydipsia, and weight loss), according to the ADA criteria (13); blood glucose levels were required to remain stable for at least 3 days to confirm successful establishment of the diabetes model.Mouse cages were changed every 2 days, and food and water were replenished promptly. Mouse body weight and blood glucose levels were measured regularly. At the experimental endpoint, mice were deeply anesthetized via intraperitoneal injection of pharmaceutical-grade pentobarbital sodium at a dose of 150 mg/kg (3× the standard anesthetic dose). The depth of anesthesia was confirmed by the absence of a pedal withdrawal reflex in response to toe pinch. Subsequently, euthanasia was performed by cervical dislocation.

#### Sample collection and processing

After the experiment, the mice were anesthetized and euthanized. Orbital blood was collected and serum was isolated for biochemical analysis. Tissue samples from the aorta and aortic root were rapidly isolated. Some were flash-frozen in liquid nitrogen for RNA and protein analysis, while others were fixed in 4% paraformaldehyde for histopathological analysis.

#### Serum biochemical assays

Mouse serum samples were analyzed using a commercial kit from Nanjing Jiancheng to measure levels of TG, TC, HDL-C, LDL-C, Interleukin-6 (IL-6), Tumor Necrosis Factor-α (TNF-α), and Interleukin-1β (IL-1β), and differences among the groups were compared.

#### Histopathological analysis

Aortic tissues were processed and subjected to hematoxylin-eosin (HE) staining and Oil Red O staining, respectively. Plaque area and lipid deposition area were measured using ImageJ software, and the ratio of plaque area to vascular lumen area was calculated.

#### Immunohistochemistry

Immunohistochemistry was used to detect ADSL (Sanying Biotech, diluted 1:100), LGALS3 (Hua’an Bio, dilution ratio 1:500), MGAT1 (Sanying Bio, dilution ratio 1:200), and VSIG4 (Hua’an Bio, dilution ratio 1:200) in aortic tissue, and semi-quantitative analysis was performed using ImageJ software.

#### RT-qPCR

Total RNA was extracted from aortic tissue using a kit from YEASEN. After assessing the concentration and purity, reverse transcription was performed to obtain cDNA. Real-time quantitative PCR was conducted using the SYBR Green method. ACTB was used as the internal reference gene, and the relative expression levels of the target genes were calculated using the 2^(−ΔΔCt) method. Specific primers for the target genes (ADSL, MGAT1, LGALS3, VSIG4) and the internal reference gene β-actin were designed using NCBI and PrimerBank software. After verifying their specificity via NCBI BLAST, the primers were synthesized by Geneworks.The primers used for RT-qPCR detection were as follows:

β-actin forward primer: 5′-CATTGCTGACAGGATGCAGAAGG-3′.

β-actin reverse primer: 5′-TGCTGGAAGGTGGACAGTGAGG-3′.

ADSL forward primer: 5′-GGATCACCAGAAGGTGGAGC-3′.

ADSL reverse primer: 5′-TGTGCACCGATGCTCCTAAG-3′.

MGAT1 forward primer: 5′-TGGATCTGGAGCCGGGAG-3′.

MGAT1 reverse primer: 5′-CCCTGCAGTCTGCTTCTTCA-3′.

LGALS3 forward primer:5′-AGACAGCTTTTCGCTTAACGA-3′.

LGALS3 reverse primer: 5′-GGGTAGGCACTAGGAGGAGC-3′.

VSIG4 forward primer:5′-CCTGGGCCACCTAATAGTGC-3′.

VSIG4 reverse primer: 5′-GCCTCTCAGGGGATCATAGAT-3′.

#### Western blot

Total proteins were extracted from aortic tissue using a kit from YEASEN. After quantification by the BCA method, the proteins were subjected to SDS-PAGE electrophoresis and transferred to a PVDF membrane. After blocking with 5% skim milk powder, the sample was incubated with the appropriate primary antibody and HRP-labeled secondary antibody. Development was performed using the ECL chemiluminescence method, with β-actin as the internal control (Hua’an Bio, dilution ratio: 1:50,000). The target proteins were ADSL (Sanying Bio, dilution ratio: 1:1,000), LGALS3 (Hua’an Bio, dilution ratio: 1:1,000), MGAT1 (Sanying Bio, dilution ratio: 1:1,000), and VSIG4 (Hua’an Bio, dilution ratio: 1:1,000). The relative expression levels of the target proteins were analyzed using ImageJ.

#### Correlation analysis between target protein expression and serum markers

Pearson correlation analysis was used to evaluate the correlation between the normalized grayscale values of the Western blot data for the four target proteins — ADSL, MGAT1, LGALS3, and VSIG4 — and serum biochemical markers and inflammatory factor levels. All statistical analyses employed two-tailed tests, with a significance level set at α = 0.05 (p < 0.05, p < 0.01, p < 0.001).For correlation analyses involving multiple comparisons, Benjamini-Hochberg FDR correction was applied, and q < 0.05 was considered statistically significant.

## Results

### Proteomics discovery phase

#### Analysis of abundance differences

A total of 739 proteins with altered abundance were detected in Pro-DM and Pro-DA compared with Pro-NC. Pro-DM yielded 121 DAPs (35 up, 86 down) and Pro-DA yielded 134 (44 up, 90 down) (**Figures 1A–D**). Sixty-nine proteins overlapped between the two diabetic groups (**Figure 1E**), suggesting a shared molecular signature that tracks diabetes progression and vascular complication development. Thresholds and statistics are as described in Methods.

**Figure 1:**
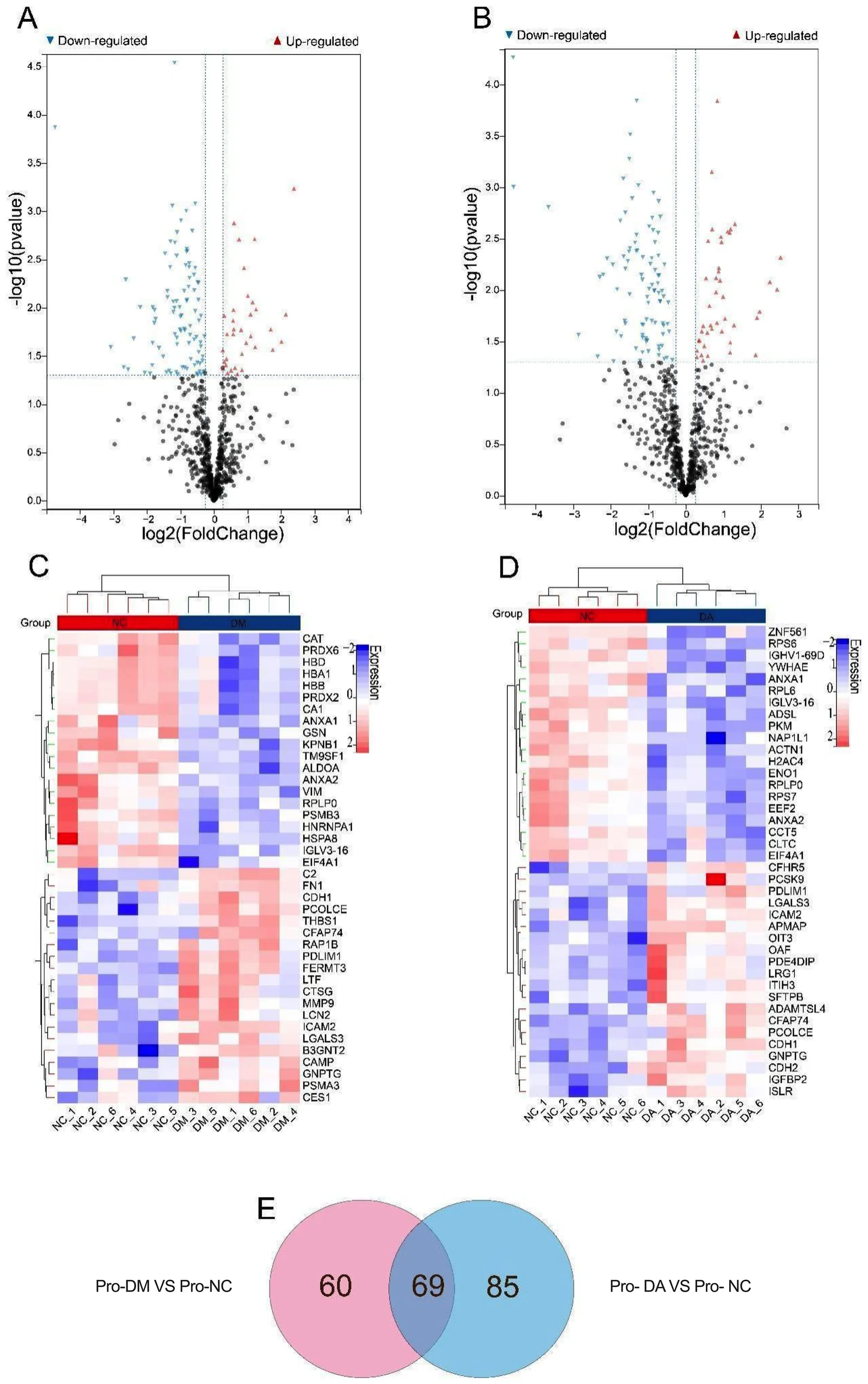
Differential abundance analysis: **(A)** volcano plot of Pro-DM versus Pro-NC; **(B)** volcano plot of Pro-DA versus Pro-NC; **(C)** heatmap of differentially abundant proteins (DAPs) between Pro-DM and Pro-NC; **(D)** heatmap of DAPs between Pro-DA and Pro-NC; **(E)** venn diagram showing the overlap of DAPs between the two comparisons: Pro-DM vs. Pro-NC and Pro-DA vs. Pro-NC.

#### Weighted protein co-expression network analysis

Given the limited sample size (n = 6 per group), WGCNA was performed as an exploratory analysis to generate hypotheses. A soft threshold of β = 6 was chosen; adjacency and topological overlap matrices were generated, and dynamic tree cutting followed by module merging produced five modules (**Figure 2A**). Eigenvector distances exceeded 0.25, indicating adequate module separation. The brown and blue-green modules were most strongly associated with the disease group (**Figures 2B–D**), and 159 proteins met hub criteria based on module membership and protein significance. Overlap with differentially abundant proteins identified 38 candidates with consistent expression trajectories from Pro-NC through Pro-DM to Pro-DA (4 upregulated, 34 downregulated) (**Figures 2E, F**), suggesting potential progression signatures. These module assignments and hub identifications should be considered preliminary and require validation in larger datasets.

**Figure 2:**
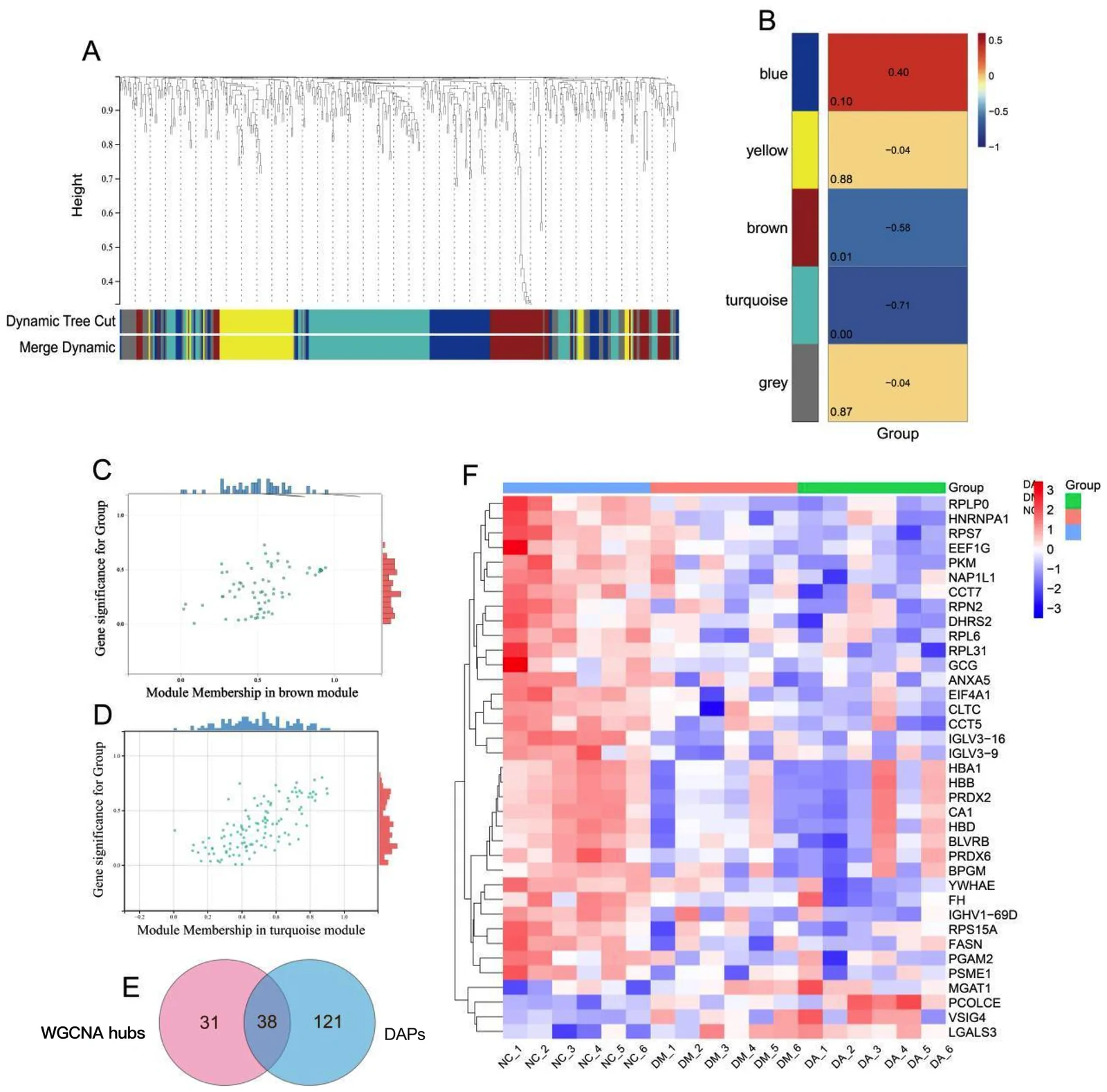
WGCNA analysis: **(A)** protein clustering dendrogram and module assignments; **(B)** heatmap showing the correlation between module eigenvectors (ME) and clinical groups; **(C)** scatter plot showing the correlation between protein significance (PS) and module membership (MM) within the brown module; **(D)** scatter plot showing the correlation between PS and MM within the blue-green module; **(E)** venn diagram showing the overlap between candidate hub proteins and differentially abundant proteins (DAPs); **(F)** heatmap of characteristic proteins.

#### Functional enrichment analysis

The 38 candidate proteins were analyzed by GO, KEGG, and GSEA. Enriched biological processes included hydrogen peroxide metabolism, oxidant detoxification, and SRP-dependent co-translational protein targeting to membranes (**Figure 3A**). Cellular components comprised cytoplasmic ribosomes, hemoglobin complexes, and the nucleus (**Figure 3B**); molecular functions encompassed peroxidase, oxidoreductase, and antioxidant activity (**Figure 3C**). KEGG analysis implicated ribosome, glucagon signaling, carbon metabolism, and N-glycosylation pathways (**Figures 3D, E**). GSEA was performed on the complete proteome ranked by differential abundance statistics. The analysis further revealed enrichment in oocyte meiosis, spliceosome, cell cycle, the tricarboxylic acid (TCA) cycle, and pyruvate metabolism (**Figure 3F**). The hemoglobin enrichment likely reflects erythrocyte contamination rather than biological relevance; however, hemolysis scores were not routinely recorded for the discovery-phase samples, which represents a limitation of this analysis.

**Figure 3:**
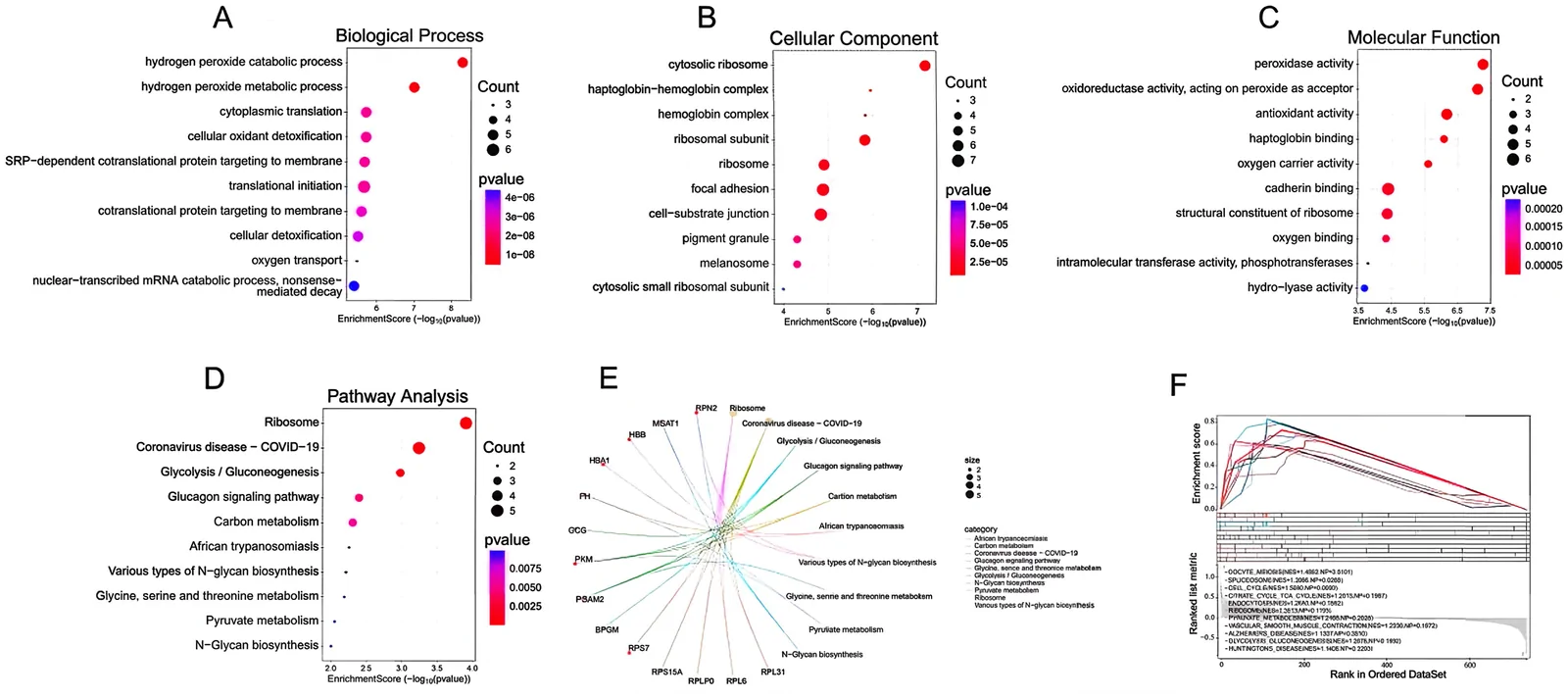
Functional enrichment analysis: **(A)** GO biological process; **(B)** GO cellular component; **(C)** GO molecular function; **(D)** KEGG pathway enrichment analysis; **(E)** KEGG pathway-protein interaction network; **(F)** gene set enrichment analysis (GSEA).

#### Protein-protein interaction (PPI) network analysis

The 38 signature proteins were mapped to STRING v11.0 and visualized in Cytoscape v3.8.0 (**Figure 4A**). Removal of 3 isolated nodes left a 35-protein network with dense interconnectivity (average degree 14.86, clustering coefficient 0.40).The high average degree and clustering coefficient may partly reflect database bias toward well-studied proteins; therefore, these results should be considered exploratory.Candidate hub proteins prioritized by both MCC and DMNC algorithms were ADSL, LGALS3, MGAT1, and VSIG4. These proteins were carried forward as candidates for downstream validation, not as confirmed biomarkers.(**Figures 4B–D**). In Pro-DA versus Pro-NC, ADSL was downregulated and LGALS3, MGAT1, and VSIG4 were upregulated, suggesting regulatory roles that require validation in independent cohorts.

**Figure 4:**
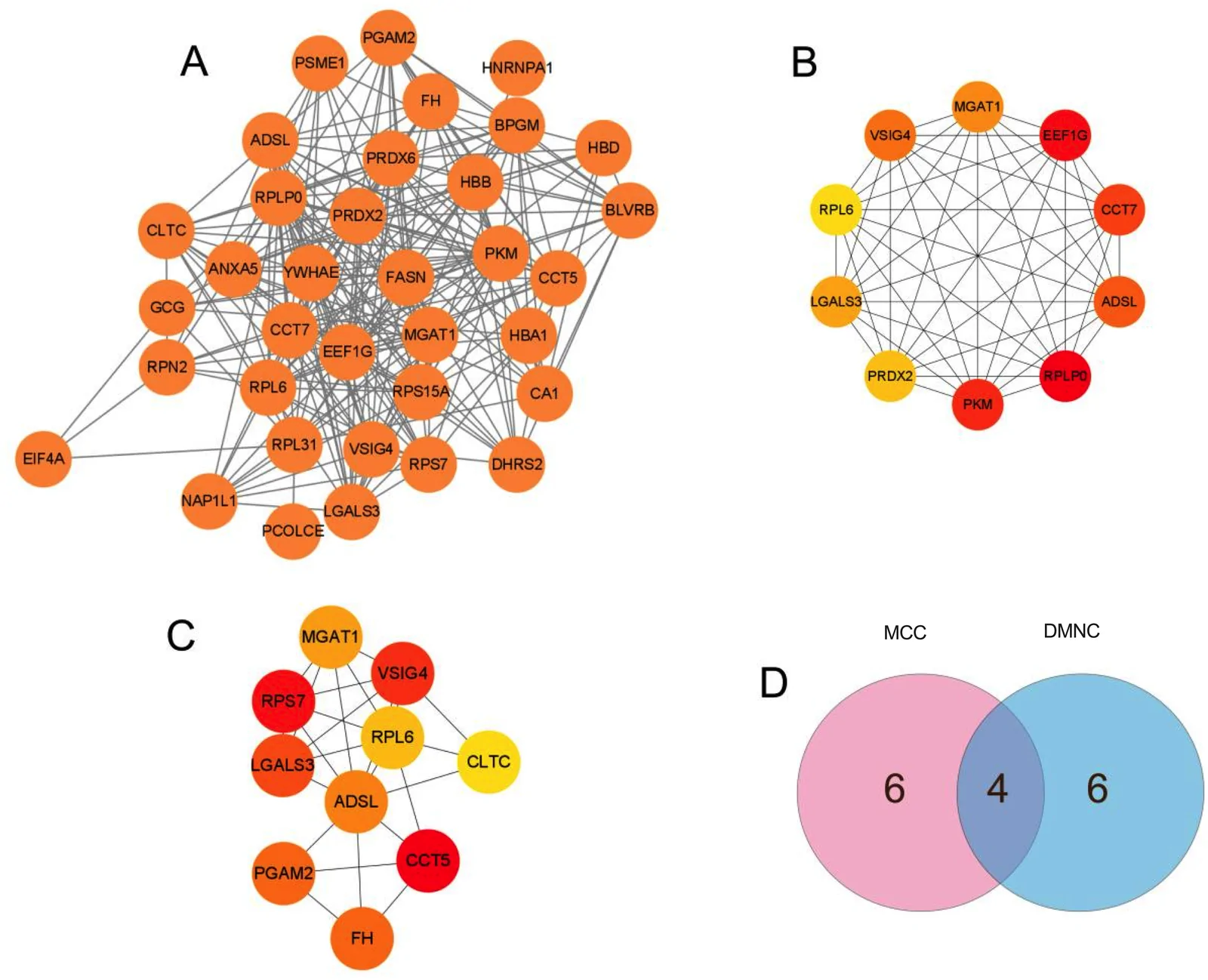
Protein-protein interaction (PPI) network analysis: **(A)** PPI network of candidate proteins; **(B)** top 10 proteins ranked by the MCC algorithm; **(C)** top 10 proteins ranked by the DMNC algorithm; **(D)** consensus candidate key proteins selected by both the MCC and DMNC algorithms.

Integration of DIA differential abundance and WGCNA prioritized 38 candidate proteins potentially associated with T2DM macrovascular complications.Enrichment analysis implicated several biological processes, including hydrogen peroxide metabolism and oxidant detoxification, vascular development and oxygen transport, ribosomal function, glucagon signaling, and carbon and amino acid metabolism. PPI network analysis further identified ADSL, LGALS3, MGAT1, and VSIG4 as candidate key proteins in the discovery cohort. These findings are preliminary and require targeted testing and external validation to establish biomarker utility and elucidate underlying mechanisms.

Because the discovery cohort was very small (n = 6 per group), all analyses in this section are exploratory and hypothesis-generating. Proteins described below were prioritized as candidate biomarkers for downstream validation, not identified as confirmed biomarkers.

### Clinical validation phase

#### Comparison of baseline characteristics

A total of 162 subjects were recruited, comprising 41 healthy controls (Pat-NC), 35 patients with T2DM alone (Pat-DM), and 86 patients with T2DM complicated by macrovascular disease (Pat-DA). None had diabetic nephropathy. The results of the intergroup comparisons are shown in **Table 1**.

**Table 1:** Comparison of demographic characteristics and clinical biochemical indicators among the three groups.

Variable	Pat-NC (n=41)	Pat-DM (n=35)	Pat-DA (n=86)	Statistics	P value
Age (years)	39.00 (34.00, 48.00)	39.00 (30.50, 46.50)	59.00 (54.00, 67.00)	H=84.36	<0.001
BMI (kg/m^2^)	24.20 (22.40, 25.70)	26.00 (24.12, 27.94)	24.37 (22.86, 25.66)	H=4.96	0.084
Systolic BP (mmHg)	121.12 ± 17.16	126.14 ± 16.25	133.94 ± 17.09	F=8.60	<0.001
Diastolic BP (mmHg)	71.00 (64.00, 80.00)	84.00 (73.50, 88.50)	81.50 (77.00, 87.00)	H=21.60	<0.001
Disease duration (years)	0.00 (0.00, 0.00)	3.00 (0.42, 7.50)	12.00 (6.00, 20.00)	H=107.98	<0.001
FBG (mmol/L)	5.14 ± 0.43	9.64 ± 3.91	8.97 ± 4.98	F=15.28	<0.001
HbA1c (%)	5.50 (5.30, 5.50)	9.60 (7.75, 10.45)	8.65 (7.20, 9.50)	H=88.38	<0.001
TC (mmol/L)	4.90 (4.40, 5.21)	4.67 (3.73, 5.73)	4.41 (3.59, 5.37)	H=5.66	0.059
TG (mmol/L)	1.12 (0.77, 1.77)	2.05 (1.28, 3.48)	1.46 (1.04, 2.20)	H=15.66	<0.001
LDL-C (mmol/L)	2.81 ± 0.77	2.56 ± 1.08	2.56 ± 1.12	F=0.87	0.421
HDL-C (mmol/L)	1.32 (1.04, 1.50)	0.90 (0.79, 1.01)	0.99 (0.82, 1.15)	H=26.59	<0.001
ADSL (ng/mL)	3.50 ± 0.52	2.84 ± 0.29	2.34 ± 0.55	F=76.11	<0.001
MGAT1 (ng/mL)	0.55 ± 0.11	0.81 ± 0.26	2.28 ± 1.06	F=85.14	<0.001
LGALS3 (ng/mL)	0.43 (0.31, 0.62)	0.53 (0.32, 0.62)	0.73 (0.50, 0.91)	H=32.16	<0.001
VSIG4 (ng/mL)	0.79 (0.58, 1.12)	1.27 (0.97, 1.45)	1.56 (1.26, 1.89)	H=38.50	<0.001
Sex (Male/Female)	16/25	20/15	56/30	χ^2^=7.70	0.021
TyG	8.50 ± 0.61	9.72 ± 1.12	9.20 ± 0.83	F=34.57	<0.001

Data from a normal distribution are expressed as mean ± standard deviation and analyzed using one-way analysis of variance (ANOVA); data from a non-normal distribution are expressed as median (interquartile range) and analyzed using the Kruskal-Wallis H test; categorical data (Sex) are expressed as frequencies (Male/Female) and analyzed using the χ^2^ test. BP, blood pressure; FBG, fasting blood glucose; HbA1c, glycated hemoglobin; TC, total cholesterol; TG, triglycerides; LDL-C, low-density lipoprotein cholesterol; HDL-C, high-density lipoprotein cholesterol; TyG, triglyceride-glucose index.

The Pat-DA group was significantly older than both the Pat-NC and Pat-DM groups (P < 0.001), with a markedly longer disease duration (P < 0.001), suggesting that age and disease duration may be important factors associated with the presence of macrovascular complications. Regarding blood pressure, systolic blood pressure was highest in the Pat-DA group, with statistically significant differences among the three groups (P < 0.001). For glycemic parameters, fasting blood glucose and HbA1c were significantly elevated in both the Pat-DM and Pat-DA groups compared with the Pat-NC group (both P < 0.001), indicating marked deterioration of glycemic control. Regarding lipid profiles, triglyceride levels were highest in the Pat-DM group, whereas HDL-C showed a gradual downward trend across the three groups; no significant differences were observed in LDL-C or total cholesterol. Overall, patients in the Pat-DA group exhibited more pronounced characteristics of advanced age, prolonged disease duration, elevated blood pressure, and dysregulation of glucose and lipid metabolism.

The TyG index differed significantly among the three groups (F = 34.57, P < 0.001). Pairwise comparisons showed that the TyG index was significantly higher in both the Pat-DM and Pat-DA groups than in the Pat-NC group (P < 0.001), and the Pat-DM group also had a higher TyG index than the Pat-DA group (P < 0.01). Additionally, prolonged disease duration in the Pat-DA group may lead to progressive β-cell dysfunction and attenuated hyperinsulinemia, which could reduce the triglyceride component of the TyG index. Furthermore, chronic low-grade inflammation in advanced diabetic macrovascular disease may alter hepatic lipid synthesis and glucose metabolism, thereby affecting TyG index calculation.

Major medication categories were recorded for both groups: statins (28.6% vs 70%), antihypertensive agents (22.9% vs 46.5%), antiplatelet drugs (0% vs 81.4%), SGLT2 inhibitors (22.9% vs 40.7%), metformin (74.3% vs 62.8%), sulfonylureas (20.0% vs 14.0%), and insulin (22.9% vs 72.1%) in Pat-DM and Pat-DA, respectively.These medications were recorded but were not included as covariates in the multivariable model because of sample size constraints and because medication use is closely linked to disease severity.

#### Distribution of macrovascular phenotypes in the Pat-DA group

Among the 86 patients in the Pat-DA group, 32 had a clinical diagnosis of coronary artery disease, 5 had ischemic stroke, and 43 had abnormal lower-extremity arterial ultrasound findings. Carotid ultrasound showed plaque formation in 64 patients, intima-media thickening without plaque in 12 patients, normal findings in 9 patients, and was not performed in 1 patient. These categories are not mutually exclusive, as patients may have more than one vascular phenotype. This distribution confirms the clinical heterogeneity of the Pat-DA group. Pooling these distinct phenotypes into a composite endpoint may dilute or confound associations with individual proteins and limits the ability to draw phenotype-specific conclusions. Therefore, the observed associations should be interpreted for prevalent macrovascular disease as a whole, and future studies with larger samples are needed to stratify by vascular bed and disease severity.

#### Changes in candidate protein expression levels

ELISA results were consistent with the proteomics discovery data (**Figures 5A–F**). ADSL expression declined with advancing disease (r = −0.699, P < 0.001), whereas MGAT1, LGALS3, and VSIG4 increased (MGAT1: r = 0.783; LGALS3 and VSIG4: both P < 0.001), with the highest levels observed in Pat-DA.

**Figure 5:**
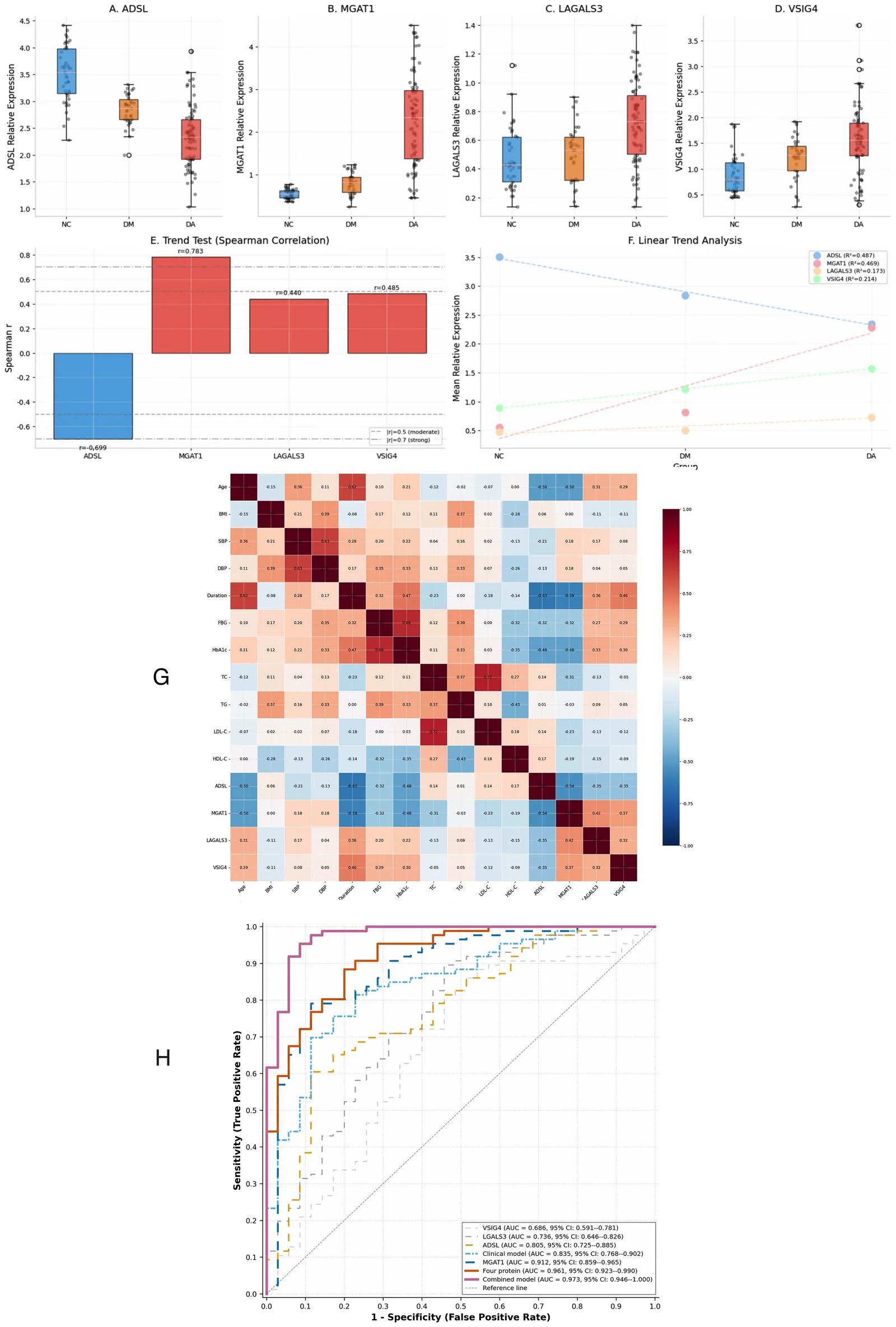
Clinical validation results. **(A)** ADSL serum levels across the three groups; **(B)** MGAT1 serum levels across the three groups; **(C)** LGALS3 serum levels across the three groups; **(D)** VSIG4 serum levels across the three groups; **(E)** trend analysis of candidate protein expression across clinical groups; **(F)** heatmap of Spearman correlation coefficients among candidate proteins; **(G)** Spearman correlation heatmap of serum candidate proteins and clinical indicators; **(H)** comparison of ROC curves for serum biomarkers and combined models in distinguishing Pat-DM from Pat-DA.Data are shown for 162 participants (41 Pat-NC, 35 Pat-DM, 86 Pat-DA). Significance markers are based on unadjusted P values. After Benjamini-Hochberg FDR correction, some correlations were no longer statistically significant; FDR-adjusted results are reported in the Results section.

#### Correlation between candidate proteins and clinical indicators

Spearman’s correlation analysis (**Figure 5G**) showed that serum ADSL levels decreased with worsening glycemic control (duration, FBG, HbA1c), while MGAT1, LGALS3, and VSIG4 increased. The inverse patterns imply that these proteins capture different facets of diabetic vascular injury rather than a single pathway. After FDR correction, ADSL, MGAT1, LGALS3, and VSIG4 remained significantly correlated with age and disease duration (q < 0.05). MGAT1 and VSIG4 also showed significant correlations with SBP, HbA1c, and several lipid parameters, whereas LGALS3 was mainly correlated with age and disease duration. Some weaker correlations did not remain significant after correction and are reported as exploratory trends.

#### Multivariate logistic regression analysis

To evaluate the independent associations of the four candidate proteins with diabetic macrovascular complications, we performed Firth penalized logistic regression, which accounts for quasi-complete separation in the standard logistic model. The model was adjusted for age, disease duration, and HbA1c. As summarized in **Table 2**, ADSL (per 1 SD increase) was significantly associated with lower odds of prevalent macrovascular complications (OR = 0.327, 95% CI: 0.069–0.972, P = 0.0199), while MGAT1 (per 1 SD increase) was significantly associated with higher odds of prevalent macrovascular complications (OR = 7.435, 95% CI: 2.471–182.763, P = 2.71×10^–6^).The extremely wide confidence interval for MGAT1 (95% CI: 2.47–182.76) reflects the limited sample size and quasi-complete separation; the point estimate should therefore not be interpreted as a precise measure of effect size, and we emphasize only the direction and statistical significance of the association.In contrast, LGALS3 (OR = 1.490, 95% CI: 0.619–4.498, P = 0.1223) and VSIG4 (OR = 1.232, 95% CI: 0.388–5.261, P = 0.2509) showed positive associations but did not reach statistical significance in the multivariable setting. These estimates should be interpreted with caution and require confirmation in larger cohorts.These associations are cross-sectional and cannot establish causality or directionality. Reverse causation remains possible.

**Table 2:** Firth penalized logistic regression analysis of serum candidate proteins associated with diabetic macrovascular complications.

Biomarker (per 1 SD increase)	OR	95% CI	P value
ADSL	0.327	0.069–0.972	0.0199
MGAT1	7.435	2.471–182.763	2.71×10^–6^
LGALS3	1.490	0.619–4.498	0.1223
VSIG4	1.232	0.388–5.261	0.2509

Adjusted for age, disease duration, and HbA1c. Due to quasi-complete separation in the standard logistic model, Firth penalized logistic regression was applied. ORs are reported with 95% profile likelihood confidence intervals. All proteins were standardized to per-1-SD increments.P values are from the penalized likelihood ratio test. The wide confidence interval for MGAT1 reflects the limited sample size and quasi-complete separation; the effect estimate should be interpreted with caution.

#### Diagnostic discrimination and incremental value of the four-protein panel

ROC curves were constructed to evaluate the discriminative ability of individual proteins and their combined panel in distinguishing Pat-DM from Pat-DA (**Figure 5H**). Individual proteins showed AUCs of 0.805 (95% CI: 0.725–0.885) for ADSL, 0.912 (95% CI: 0.859–0.965) for MGAT1, 0.736 (95% CI: 0.646–0.826) for LGALS3, and 0.686 (95% CI: 0.591–0.781) for VSIG4 (**Table 3**). The four-protein combined model yielded an AUC of 0.961 (95% CI: 0.923–0.990), significantly higher than each individual protein (all P < 0.05, DeLong test). The clinical model, comprising age, disease duration, and HbA1c, achieved an AUC of 0.835 (95% CI: 0.768–0.902). When the four-protein panel was added to the clinical model, the AUC increased to 0.973 (95% CI: 0.946–1.000), corresponding to a ΔAUC of 0.138 (Bootstrap 95% CI: 0.071–0.205; DeLong test P = 0.008), indicating that the combined model (clinical variables + four proteins) provides significant incremental discriminatory value over the clinical model alone (**Figure 5H**). These analyses reflect prevalent disease status and do not provide evidence for prediction of future cardiovascular events.

**Table 3:** Cross-validation evaluation results (5-fold stratified cross-validation).

Model	Apparent AUC (95% CI)	CV-AUC (mean ± SD)	Optimism	Calibration slope	Brier score	NRI	IDI
Single protein — ADSL	0.805 (0.725–0.885)	——	–	–	–	–	–
Single protein — MGAT1	0.912 (0.859–0.965)	——	–	–	–	–	–
Single protein — LGALS3	0.736 (0.646–0.826)	——	–	–	–	–	–
Single protein — VSIG4	0.686 (0.591–0.781)	——	–	–	–	–	–
Four-protein combined model	0.961 (0.923–0.990)	——	–	–	–	–	–
Clinical model†	0.835 (0.768–0.902)	0.812 ± 0.071	–	–	–	Ref	Ref
Clinical + protein panel	0.973 (0.946–1.000)	0.972 ± 0.033	0.001	≈1.000	0.032	0.314	0.261

^†^Clinical model includes age, disease duration, and HbA1c. NRI and IDI are reported for the clinical+protein panel versus the clinical model alone. CV-AUC was calculated from five-fold stratified cross-validation repeated 50 times. Optimism, Apparent AUC − CV-AUC. —, not applicable/not calculated.

The categorical NRI for the combined model versus the clinical model alone was 0.314 (95% CI: 0.163–0.489, P < 0.001), and the IDI was 0.261 (95% CI: 0.181–0.351, P < 0.001).These exploratory measures suggest incremental diagnostic value but should be interpreted cautiously given the cross-sectional design.Calibration of the combined model was good, with a calibration slope of approximately 1.000, calibration-in-the-large of 0.000, and Brier score of 0.032 (**Supplementary Figure S1A**). Decision curve analysis demonstrated that the combined model provided positive net clinical benefit across threshold probabilities (**Supplementary Figure S1B**).

Five-fold stratified cross-validation (repeated 50 times) yielded a mean CV-AUC of 0.812 ± 0.071 for the clinical model and 0.972 ± 0.033 for the combined model (optimism = 0.001) (**Table 3**), supporting internal stability without substantial overfitting.These results reflect internal validation within a single clinical cohort. The high AUC should be interpreted with caution, and external validation is needed to confirm generalizability.

### Animal experiments

#### General information on the mice

To provide supportive evidence for candidate protein expression, a diabetic atherosclerosis mouse model was established using a high-fat diet combined with streptozotocin. The Mod-DM and Mod-DA groups exhibited hyperglycemia, weight gain, and dyslipidemia throughout the study. Post-STZ blood glucose remained ≥ 11.1 mmol/L, confirming successful diabetes induction. The Mod-DA group showed significantly higher body weight, blood glucose, and lipid levels than both Mod-NC and Mod-DM during early high-fat feeding.

During the initial 5-week high-sugar, high-fat feeding, Mod-DM and Mod-DA demonstrated accelerated weight gain versus Mod-NC (regular diet), presenting an obese phenotype (**Figure 6A**). At week 5, after STZ injection, body weight ranked Mod-NC < Mod-DM < Mod-DA. Post-STZ, all groups continued gaining weight, but growth rates diverged: Mod-NC stable, Mod-DM and Mod-DA slowed. Throughout 24 weeks, Mod-DM and Mod-DA maintained higher body weights than Mod-NC, consistent with early T2DM features.

**Figure 6:**
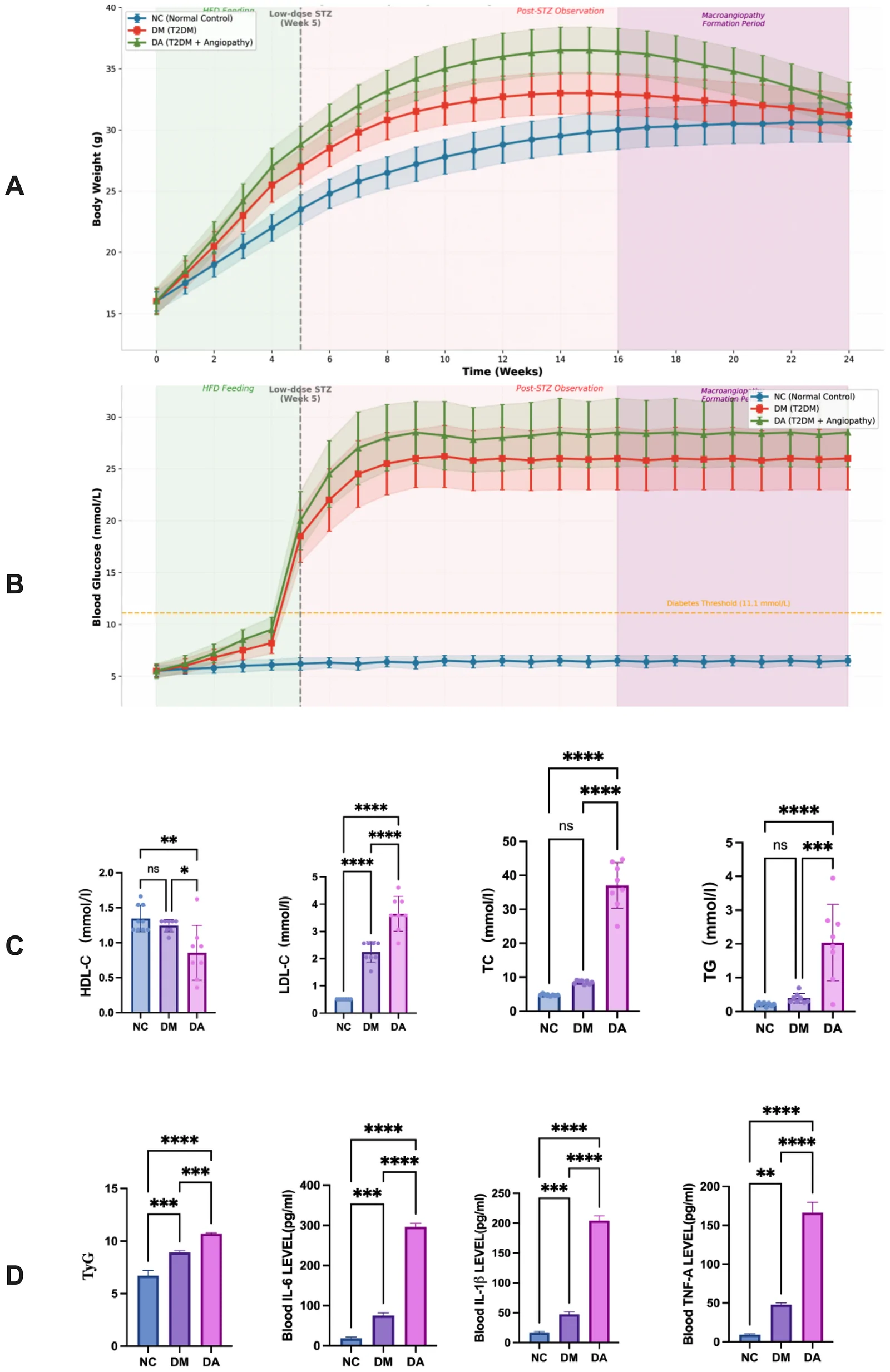
**(A)** Body weight changes in mice. NC, normal control group; DM, type 2 diabetes group; DA, type 2 diabetes with macrovascular complications group; **(B)** Trends in blood glucose levels across the three groups; **(C, D)** Comparison of biochemical parameters among the three groups of mice. **(A–D)** Data are shown as mean ± SD. n = 10 (Mod-NC), 9 (Mod-DM), and 8 (Mod-DA) mice per group. Missing data were due to death before the study endpoint, as described in Methods. *P < 0.05, **P < 0.01, ***P < 0.001, ****P < 0.0001, and ns = not significant.

Fasting blood glucose trended upward during initial 5-week feeding (week 5: Mod-NC 6.2 ± 0.6, Mod-DM 8.2 ± 1.0, Mod-DA 9.5 ± 1.2 mmol/L) (**Figure 6B**). Post-STZ, Mod-DM and Mod-DA glucose exceeded 11.1 mmol/L within one week, peaking at weeks 7–8 (Mod-DM 26.0 ± 2.8, Mod-DA 28.5 ± 3.0 mmol/L), then maintaining stable hyperglycemia. Mod-NC glucose remained normal (week 12: 6.5 ± 0.5 mmol/L). Persistently higher Mod-DA glucose may reflect combined high-fat diet and atherosclerosis modeling. These findings support successful T2DM induction with insulin resistance and β-cell dysfunction.

Biochemical analysis showed that Mod-DM and Mod-DA exhibited significant lipid metabolism disturbance versus Mod-NC (**Figure 6C**). HDL-C was markedly reduced in Mod-DM and Mod-DA, more pronounced in Mod-DA. LDL-C, TC, and TG showed progressive elevation, greatest in Mod-DA. Diabetes-associated lipid metabolism disturbance was more severe in Mod-DA.

Mod-DM and Mod-DA showed significantly elevated TyG index and serum IL-6, IL-1β, and TNF-α versus Mod-NC (**Figure 6D**). Mod-NC maintained the lowest inflammatory parameters. Diabetes was associated with increased insulin resistance and systemic inflammation, with further TyG index, IL-6, and TNF-α elevation in Mod-DA.

#### Pathological lesions of the aorta

Oil Red O staining (**Figures 7A, B**) showed heavy lipid loading in Mod-DA; the plaque-to-lumen ratio was higher than in Mod-NC or Mod-DM.

**Figure 7:**
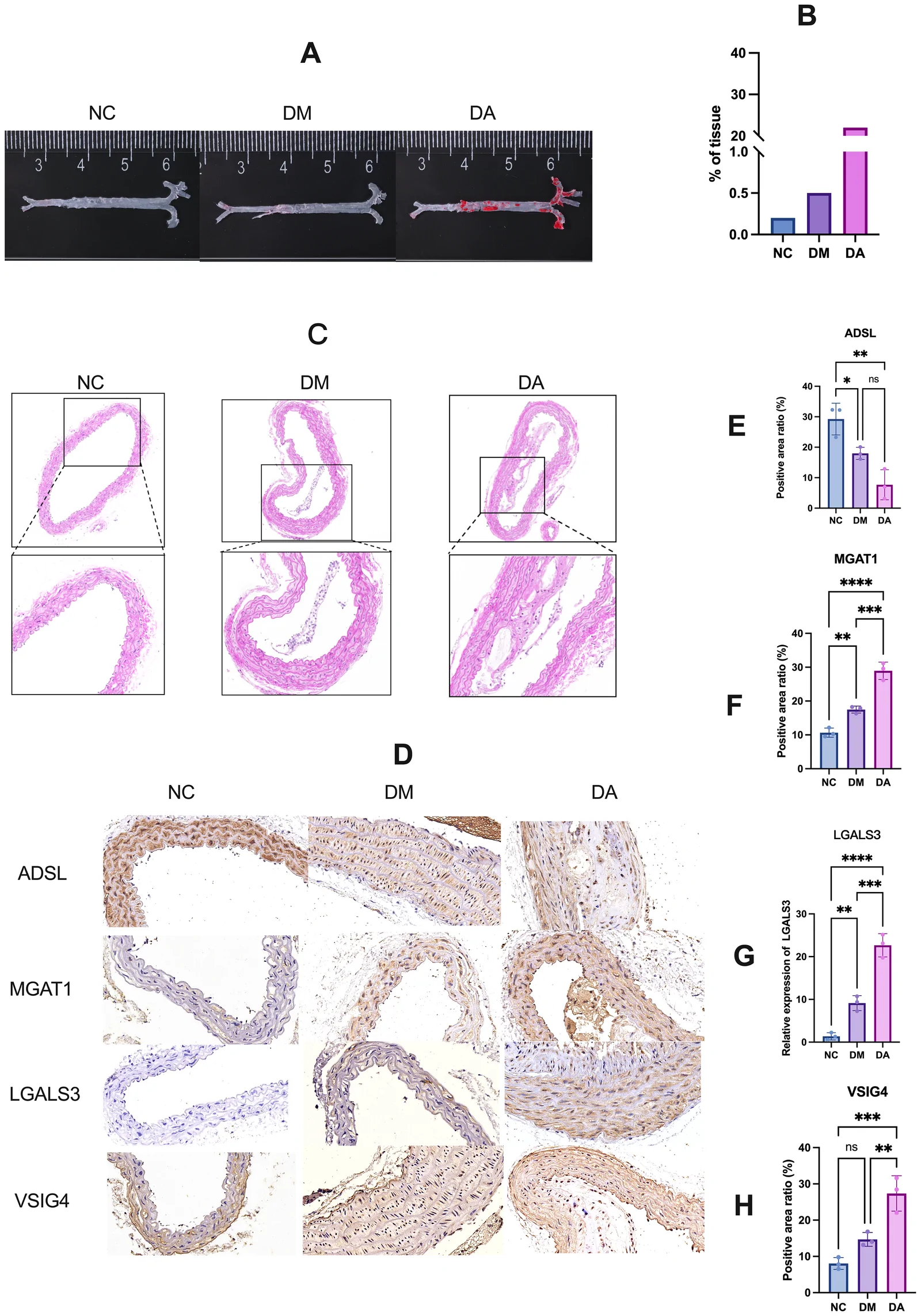
Pathological staining results of the aortas in the three groups of mice. **(A)** Oil Red O staining of the aorta; **(B)** Quantitative analysis of plaque area/lumen area. *P < 0.05, **P < 0.01; **(C)** Hematoxylin and eosin (HE) staining of the aorta; **(D)** Immunohistochemical detection of target protein expression in aortic tissue; **(E–H)** Immunohistochemical results. *P < 0.05, **P < 0.01, ***P < 0.001, ****P < 0.0001, and ns = not significant. Representative images are shown from three randomly selected mice per group (n = 3 per group).

HE sections (**Figure 7C**) fell into three clear grades. Mod-NC vessels appeared normal: open lumen, thin intima, and clean wall. Mod-DM had thickened intima with foam cells and scattered subendothelial inflammation, plus mild narrowing — consistent with early atheroma. Mod-DA showed the most severe changes: lumen severely compromised by complex plaques with fibrous caps, necrotic cores, cholesterol clefts, calcium deposition, and neovascularization; the media was also disorganized.

#### Localization and expression of target proteins in aortic tissue

Immunohistochemistry revealed an inverse expression pattern for ADSL versus the other three candidate proteins in aortic tissue (**Figures 7D–H**). ADSL was abundant in Mod-NC, reduced in Mod-DM, and sparse in Mod-DA. In contrast, LGALS3, MGAT1, and VSIG4 were strongly expressed in Mod-DA plaques and vascular walls, with lower levels in Mod-DM and Mod-NC.

#### RT-qPCR validation

RT-qPCR confirmed the expression trends observed at the protein level (**Figure 8G**). ADSL mRNA decreased progressively across the three groups, whereas MGAT1, LGALS3, and VSIG4 mRNAs were significantly upregulated in both diabetic groups, with the highest levels in Mod-DA (all P < 0.01 vs. Mod-NC). These findings indicate that the differential expression of the four candidate genes is regulated at the transcriptional level.

**Figure 8:**
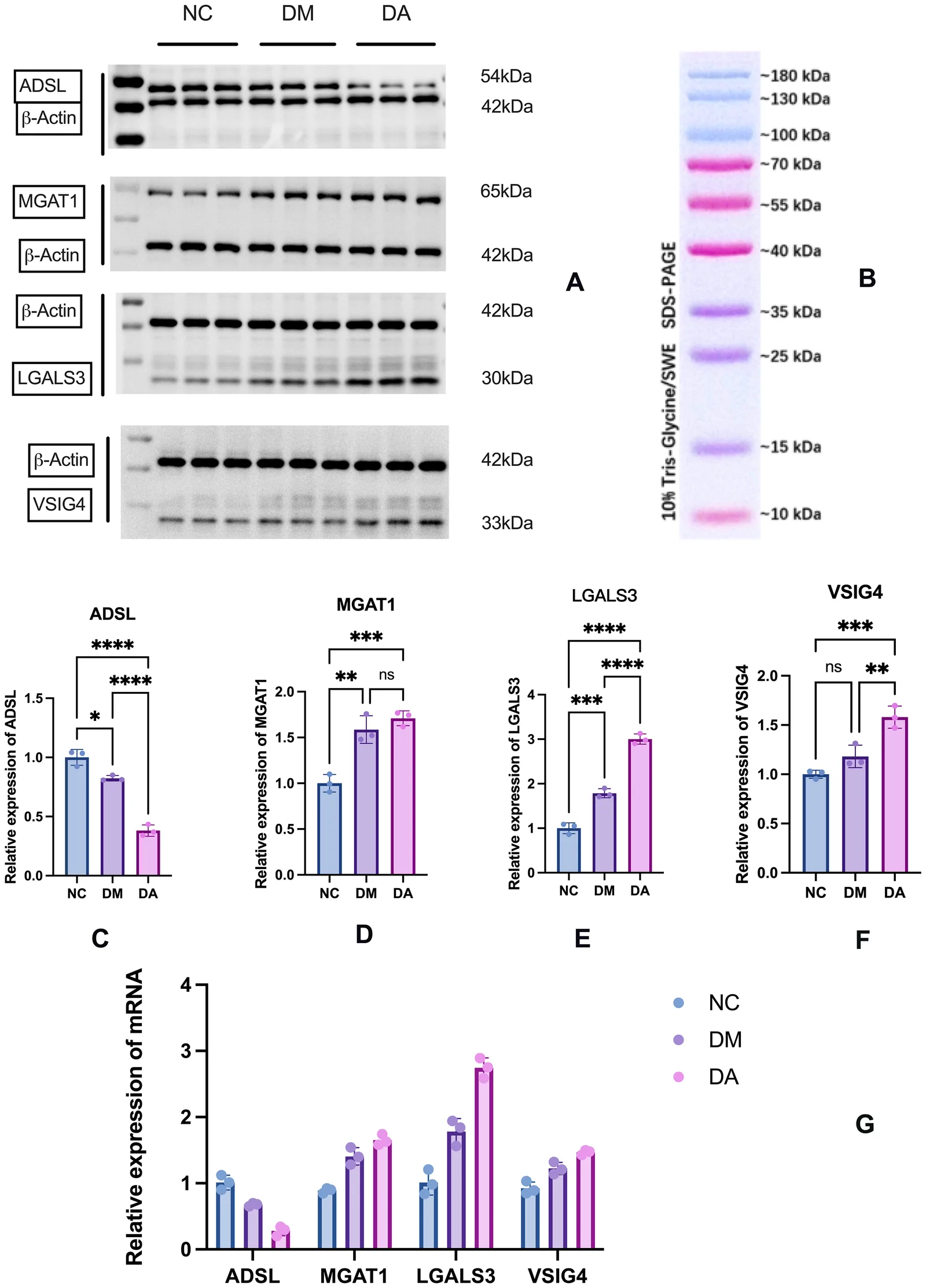
Western blot and RT-qPCR analysis of target protein expression levels in aortic tissue. **(A)** Representative Western blot bands of ADSL, MGAT1, LGALS3, and VSIG4 in aortic tissues from the three groups; β-actin was used as the loading control. **(B)** Protein band patterns; **(C–F)** Relative expression levels of candidate proteins; Western blot analysis was performed on aortic tissues from three randomly selected mice per group (n = 3 per group). **(G)** Relative expression levels of the four proteins at the mRNA level. *P < 0.05, **P < 0.01, ***P < 0.001, ****P < 0.0001, and ns = not significant. n = 10 (Mod-NC), 9 (Mod-DM), and 8 (Mod-DA) mice per group.

#### Western blot validation

Western blot analysis supported the findings at the protein level (**Figures 8C–F**). ADSL was lowest in Mod-DA, while LGALS3, MGAT1, and VSIG4 were highest in Mod-DA. These patterns are consistent with clinical serum data and proteomics discovery results, although differences between groups may partly reflect genetic background (see Limitations).

#### Correlation between target protein expression and serum markers

To investigate the association between the four candidate proteins and metabolic as well as inflammatory changes in diabetic atherosclerosis, Western blot band intensities were correlated with serum lipids (TC, TG, LDL-C, HDL-C) and cytokines (IL-6, TNF-α, IL-1β) (**Figure 9A**).

**Figure 9:**
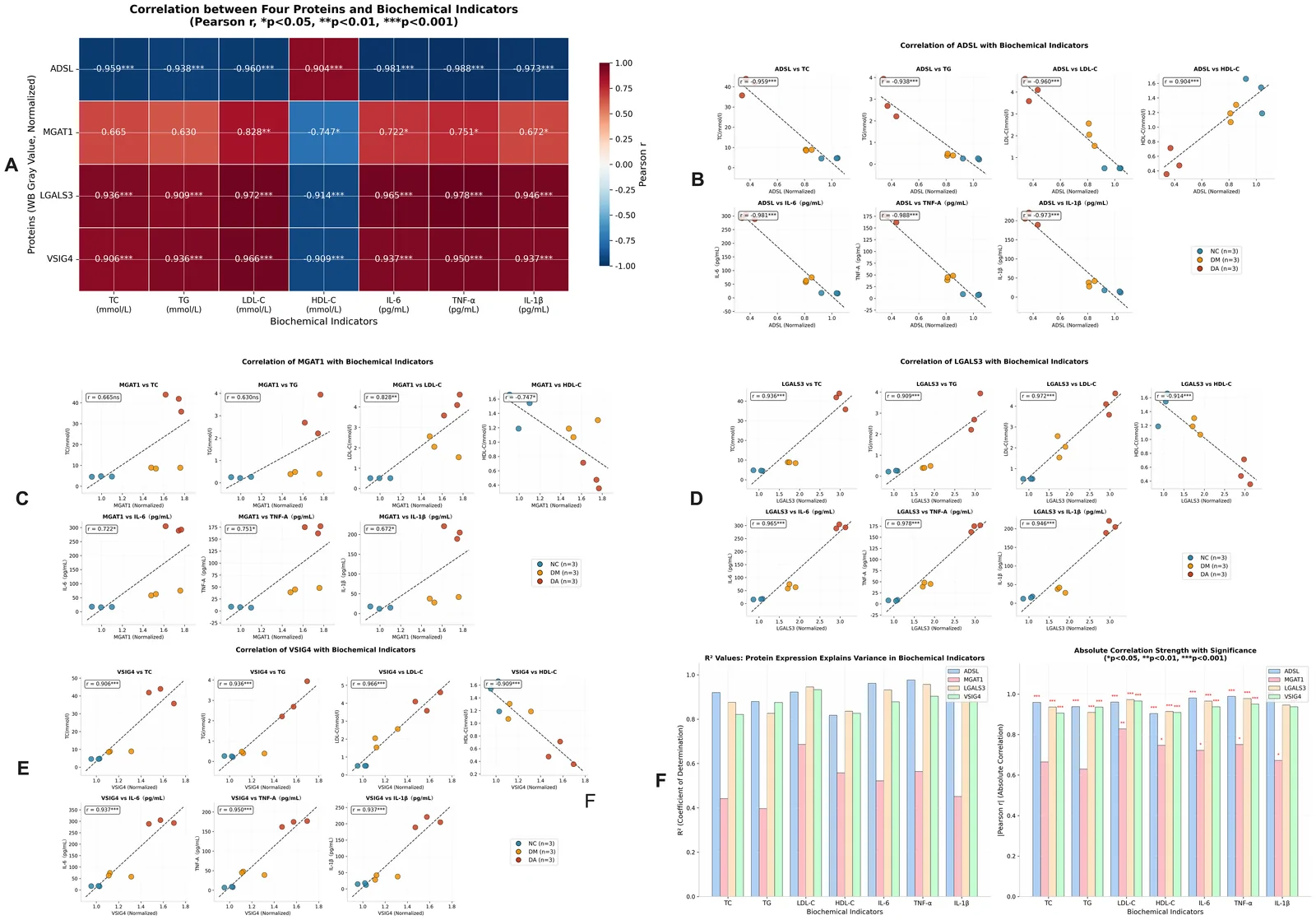
Correlation analysis between target protein expression levels and serum biochemical and inflammatory markers. **(A)** Correlation heatmap; **(B–E)** Scatter plots of candidate proteins versus the 7 markers; **(F)** Comparative bar chart of R^2^ and R values.Correlation analysis using data from 9 mice (3 per group). Significance markers are based on unadjusted P values. After Benjamini-Hochberg FDR correction, some correlations were no longer statistically significant; FDR-adjusted results are reported in the Results section.

ADSL correlated inversely with TC, TG, LDL-C, and all three cytokines, while correlating directly with HDL-C (all P < 0.001). MGAT1 tracked with LDL-C (P < 0.01) and cytokines (all P < 0.05), though not with TC or TG. LGALS3 and VSIG4 rose and fell with TC, TG, LDL-C, and cytokines, moving opposite to HDL-C (all P < 0.001). The strongest linear fits were observed for LGALS3–LDL-C (R^2^ = 0.945) and ADSL–TNF-α (R^2^ = 0.977) (**Figures 9B–E**).

These patterns point to distinct mechanistic roles in lipid metabolism and inflammation. The clinical-animal discrepancy for MGAT1, which was the best ROC performer (AUC = 0.912) yet showed the weakest Pearson correlations, reflects a fundamental methodological difference. ROC evaluates categorical discrimination between Pat-DM and Pat-DA, whereas Pearson evaluates continuous covariation with biochemical markers. ADSL, LGALS3, and VSIG4 showed stronger correlations with metabolic and inflammatory markers, whereas MGAT1’s diagnostic performance may reflect threshold-based discrimination rather than continuous covariation with these markers (**Figure 9F**). After FDR correction, ADSL, LGALS3, and VSIG4 remained significantly correlated with all seven biochemical indicators (q < 0.05). MGAT1 remained significantly correlated with LDL-C, HDL-C, IL-6, and TNF-α, whereas its correlations with TC, TG, and IL-1β did not pass the FDR threshold.

## Discussion

### Principal findings

In this study, we screened the plasma proteome, validated candidate proteins in an independent clinical cohort, and supported the findings in a mouse model. In an exploratory discovery phase, four candidate proteins were prioritized: ADSL expression decreased across clinical groups, while MGAT1, LGALS3, and VSIG4 expression increased. Because the discovery cohort was very small (n = 6 per group), these findings should be considered hypothesis-generating.All four proteins correlated with disease duration, metabolic control parameters, and inflammatory markers. The four-protein combined model outperformed any single marker (AUC = 0.961), consistent with the notion that each protein reflects a distinct pathological process: metabolism (ADSL), glycosylation (MGAT1), inflammation (LGALS3), and immune regulation (VSIG4).

In the Firth penalized regression model adjusting for age, disease duration, and HbA1c, ADSL (OR = 0.327, P = 0.0199) and MGAT1 (OR = 7.435, P = 2.71×10^–6^) demonstrated independent associations with prevalent macrovascular disease. We hypothesize that the attenuation of LGALS3 and VSIG4 in the multivariable model may reflect cumulative metabolic and inflammatory burden rather than a lack of biological relevance. Both proteins are closely correlated with age and disease duration, which are inseparable from the progression of T2DM. Thus, their lack of independent significance may indicate that their diagnostic information is largely captured by the cumulative disease burden, rather than that they are biologically unimportant. This hypothesis is supported by their consistent upregulation in the animal model, where the influence of age and disease duration is minimized. Nevertheless, this interpretation remains speculative and requires further validation.Furthermore, the lack of independent statistical significance for LGALS3 and VSIG4 may also reflect limited statistical power and collinearity with age and disease duration, rather than a lack of diagnostic relevance. Both proteins showed moderate discriminative ability in univariable ROC analysis, and their consistent upregulation in the animal model supports their biological relevance. Larger studies are needed to determine their independent diagnostic value.

The combined model (clinical variables + four proteins) demonstrated significant improvement over the clinical model alone (ΔAUC = 0.138, P = 0.008; NRI = 0.314, P < 0.001; IDI = 0.261, P < 0.001), underscoring the complementary value of the protein panel in a multi-marker strategy. The good calibration (Brier score = 0.032, calibration slope ≈ 1.000) and internal validation (CV-AUC = 0.972 ± 0.033, optimism = 0.001) support the robustness of these findings.

### Background and mechanisms of diabetic macrovascular complications

Macrovascular complications represent the leading cause of mortality in patients with T2DM. The pathogenesis involves insulin resistance, metabolic dysfunction, chronic inflammation, and endothelial injury. Although pharmacological management of glycemia, blood pressure, and lipids has improved, atherosclerosis remains a major cause of death in this population. There remains a critical need for biomarkers that facilitate diagnostic stratification and individualized management in patients with prevalent macrovascular complications.

Insulin resistance is the core pathophysiological basis of T2DM and a major factor driving the development of macrovascular complications (14). The TyG index, as a simple surrogate marker for insulin resistance, has been used to assess metabolic abnormalities and cardiovascular risk (15). This study measured the TyG index to evaluate the relationship between insulin resistance, the expression of candidate proteins, and the development of macrovascular complications in T2DM. Dyslipidemia is also a major driver of atherosclerosis; patients with T2DM often exhibit elevated triglycerides (TG), reduced HDL-C, and an atherogenic lipoprotein profile, which promote lipid deposition in the vascular wall and plaque formation (16).

Furthermore, chronic hyperglycemia can exacerbate oxidative stress and endothelial dysfunction through mechanisms such as the formation of advanced glycation end products (AGEs), activation of the protein kinase C (PKC) pathway, abnormalities in the polyol pathway, and activation of NADPH oxidase (17). Inflammatory responses also play a crucial role in the progression of diabetic macrovascular complications; pro-inflammatory factors such as IL-6, IL-1β, and TNF-α promote monocyte recruitment, macrophage activation, and plaque inflammation (18–20). This study measures glucose and lipid metabolism and inflammation-related markers in clinical and animal experiments and further analyzes their relationships with candidate proteins, aiming to preliminarily explore the potential significance of these proteins in T2DM macrovascular complications from both metabolic and inflammatory perspectives.

### ADSL

ADSL is a key enzyme in purine metabolism. It participates in both *de novo* purine synthesis and the purine nucleotide cycle, playing a crucial role in maintaining cellular energy homeostasis and nucleotide metabolic balance. Previous research on ADSL has primarily focused on ADSL deficiency syndrome (21), neurological abnormalities, and tumor metabolic reprogramming (colorectal (22), breast (23), liver (24), and prostate (25)); however, its role in diabetic vascular complications has not yet been systematically studied. ADSL was downregulated in the proteomics screen, and this decrease was confirmed in both the validation cohort and the mouse model. In patients, lower ADSL was associated with longer disease duration, higher HbA1c, and higher fasting glucose (all P < 0.05), indicating that ADSL expression decreases as glucose control deteriorates over time.

In the Mod-DA aorta, ADSL expression was also reduced and inversely correlated with lipid and inflammatory markers. ADSL catalyzes two steps in purine biosynthesis: the conversion of SAICAR to AICAR and the conversion of SAMP to AMP. Reduced ADSL activity would be expected to impair purine nucleotide synthesis, potentially leading to insufficient ATP regeneration and compromised energy supply to vascular endothelial cells (26). Impaired energy metabolism in endothelial cells promotes the release of inflammatory factors and vascular remodeling processes (27). Additionally, decreased ADSL may lead to accumulation of its substrates (SAICAR and SAMP), the functional consequences of which in vascular tissue remain to be determined. Disruption of intracellular nucleotide homeostasis may also affect endothelium-dependent vasodilation (28). Furthermore, purine metabolic disorders may affect the function of vascular smooth muscle cells, promoting their proliferation and migration, thereby accelerating the atherosclerotic process (29). The precise mechanistic link between reduced ADSL and diabetic vascular damage warrants further investigation through targeted knockout and rescue experiments.

### MGAT1

MGAT1 is one of the key enzymes in the N-glycosylation pathway. It is involved in the synthesis of complex N-glycan chains and plays an important role in protein folding, receptor stability, cell adhesion, and signal transduction (30). In recent years, abnormal glycosylation has been found to be closely associated with insulin resistance, chronic inflammation, and cardiovascular disease (31–33). This study found that MGAT1 levels were significantly elevated in the Pat-DA group and demonstrated the highest individual diagnostic performance among the candidate proteins. Clinical validation results show that MGAT1 is positively correlated with clinical group classification and disease duration, and ROC analysis indicates favorable diagnostic performance in distinguishing between Pat-DM and Pat-DA. These findings suggest that MGAT1 may be a candidate biomarker for macrovascular complications of T2DM, although its incremental value beyond conventional clinical indicators requires further evaluation.

Based on existing research, abnormalities in N-glycosylation may impair the structural stability and signal transduction of the insulin receptor, along with various cell membrane receptors (31). Elevated MGAT1 expression may alter the glycosylation status of cell surface proteins, thereby affecting insulin signaling, endothelial function, and the migration as well as proliferation of vascular smooth muscle cells. Furthermore, the complex N-glycan chains mediated by MGAT1 may also interact with proteins of the galectin family, thereby influencing cell membrane receptor clustering and inflammatory signaling (32). In this study, MGAT1 was positively correlated with LGALS3 and VSIG4, suggesting a potential association between MGAT1 and glycosylation, inflammatory responses, and immune regulation.

The apparent discrepancy between the strong ROC performance of MGAT1 and its moderate correlations with individual biochemical markers likely reflects methodological differences: ROC evaluates group discrimination, whereas correlation assesses continuous covariation. Biologically, MGAT1 may reflect an overall N-glycosylation state rather than tracking any single lipid or cytokine linearly. In both the clinical cohort and the animal model, MGAT1 was significantly correlated with several metabolic and inflammatory markers, but not all, and the correlation strengths were moderate. This pattern is consistent across species and supports the biological relevance of MGAT1 while suggesting that its diagnostic value may rely on threshold-based discrimination.

### LGALS3

LGALS3 encodes galectin-3, a β-galactoside-binding protein that is widely involved in processes such as inflammatory responses, fibrosis, macrophage activation, and immune regulation (34). Previous studies have shown that galectin-3 is closely associated with insulin resistance, chronic low-grade inflammation, atherosclerotic plaque formation, and the risk of cardiovascular events (35–37). This study found that LGALS3 expression was elevated in the Pat-DA group and was positively correlated with clinical group classification, glucose metabolism markers, and inflammation-related markers. In animal experiments, LGALS3 expression was increased in the aortic plaque region and the vascular wall of the Mod-DA group, further supporting its potential involvement in inflammation-related responses associated with diabetic atherosclerosis.

Previous studies have suggested that galectin-3 promotes macrophage activation and the release of pro-inflammatory cytokines. It may also contribute to plaque formation and progression through multiple mechanisms, including activation of the NLRP3 inflammasome, involvement in AGE-related pathways, and modulation of vascular smooth muscle cell function (38, 39). Therefore, the results of this study are consistent with previous literature reports, suggesting that elevated LGALS3 levels may reflect inflammatory activation and plaque-related immune responses in diabetic macrovascular disease.

### VSIG4

VSIG4 belongs to the B7 family of molecules and is primarily expressed on the surface of tissue-resident macrophages, where it participates in complement regulation, inhibition of T-cell activation, and regulation of the immune state of macrophages (40, 41). Previous studies suggest that VSIG4 is associated with chronic inflammation, immune tolerance, and tissue repair processes (42); however, its role in diabetic macrovascular complications remains unclear. This study found that VSIG4 expression was elevated in the DA group and showed an upward trend across clinical groups. Clinical correlation analysis revealed that VSIG4 was positively correlated with disease duration and glucose metabolism indices; in animal experiments, VSIG4 expression was enhanced in aortic tissue from the Mod-DA group and was associated with lipid profile and inflammatory cytokine levels. These results suggest that elevated VSIG4 levels may reflect alterations in macrophage-related immune regulation and the chronic inflammatory state in diabetic macrovascular disease.

From a mechanistic perspective, VSIG4 may be involved in the regulation of the complement alternative pathway, macrophage polarization, and T-cell immune responses. Its elevated expression may represent a compensatory response to the chronic inflammatory state, or it may be associated with an imbalance in the immune microenvironment and plaque progression.

### Four-protein combined model

In this cross-sectional clinical cohort, the four-protein combined model showed strong diagnostic discrimination and outperformed any single marker (AUC = 0.961). This model was internally validated by repeated cross-validation. Its biological plausibility is further supported by the animal model, in which all four proteins were consistently dysregulated in aortic tissue and correlated with metabolic and inflammatory markers.This is consistent with the notion that each protein reflects a distinct pathological process: ADSL in purine metabolism and energy homeostasis; MGAT1 in N-glycosylation and insulin signaling; LGALS3 in macrophage-driven inflammation; and VSIG4 in immune regulation. Together, they characterize diabetic atherosclerosis from four complementary perspectives.While external validation in independent prospective cohorts remains necessary, these findings support the four-protein panel as a promising candidate for diagnosing prevalent macrovascular complications in T2DM.

The four-protein panel was not directly compared with established cardiovascular biomarkers such as hs-CRP, oxLDL, or the ApoB/ApoA1 ratio, as these were not measured in the present cohort. These markers reflect distinct aspects of inflammation, oxidative stress, and lipoprotein balance, and each has recognized value in cardiovascular risk assessment. Future studies should directly compare the four-protein panel with these established markers to determine whether it provides additive or complementary diagnostic information.

### Strengths and limitations

This study has the following strengths:

1. The research approach, which combines proteomic screening, clinical validation, and animal experiments, provides multi-level evidence for the candidate biomarkers.
2. The combined use of WGCNA (performed as an exploratory analysis given the limited sample size) and PPI network analysis to identify key proteins provides complementary candidate screening, although these findings require validation in larger independent datasets.
3. Systematic evaluation of diagnostic performance through ROC analysis and cross-validation provides preliminary evidence of model stability.

However, this study has several important limitations that must be acknowledged:

1. This was a single-center, cross-sectional study with a relatively limited sample size. The cross-sectional design precludes causal and temporal inference; the observed associations reflect prevalent macrovascular complications rather than incident events, and reverse causation cannot be excluded. The Pat-DA group was older and had longer disease duration than the Pat-NC group; although multivariate adjustment was performed, residual confounding, including smoking status, which was not systematically available for all participants, cannot be excluded.In addition, the discovery cohort was a single-center convenience sample with stringent group definitions, which may introduce potential selection and spectrum bias.
2. The proteomics discovery cohort comprised only 18 subjects (n = 6 per group). Because of this very small sample size, WGCNA was exploratory, and no formal sensitivity or stability analysis was performed, as such analyses would be severely underpowered and potentially misleading. Module assignments and hub protein identification therefore require confirmation in larger independent datasets.
3. The Pat-DA group included heterogeneous macrovascular phenotypes. Pooling these phenotypes may introduce clinical heterogeneity and residual confounding and may mask phenotype-specific associations. Because of the limited sample size, phenotype-specific sensitivity analyses were not statistically meaningful. Future studies should stratify patients by vascular bed and disease severity.
4. The Mod-DA group used an ApoE−/− genetic background, while the Mod-NC and Mod-DM groups used C57BL/6J. Group differences therefore simultaneously reflect diabetes, atherosclerosis, and genetic background effects. Animal results should be interpreted as supportive rather than definitive evidence that the four proteins are specifically caused by diabetic macrovascular disease.
5. In-depth mechanistic experiments, including loss-of-function, gain-of-function, knockout, rescue, or pathway intervention studies, have not yet been conducted. Therefore, the biological roles of ADSL and MGAT1 in diabetic atherosclerosis remain hypothetical. Future cellular and molecular studies are required to validate the underlying mechanisms.

Future studies should include prospective longitudinal multicentre cohorts with serial sampling before and after the onset of macrovascular complications. Such studies are needed to determine the temporal ordering of these protein changes, to assess whether they can predict incident events, and to clarify whether they have true early biomarker potential. Mechanistic experiments using loss-of-function, gain-of-function, knockout, or rescue approaches are also warranted to define the causal pathways linking these proteins to diabetic macrovascular injury.

## Conclusion

ADSL and MGAT1 were independently associated with prevalent macrovascular disease in this single-center cross-sectional cohort. The four-protein panel (ADSL, MGAT1, LGALS3, VSIG4) demonstrated significant incremental diagnostic value over age, disease duration, and HbA1c (ΔAUC = 0.138, P = 0.008; NRI = 0.314, P < 0.001; IDI = 0.261, P < 0.001), with good calibration and internal stability. However, LGALS3 and VSIG4 did not reach statistical significance in the Firth penalized regression model. These findings suggest that ADSL and MGAT1 may serve as candidate biomarkers, while the combined panel offers complementary value for identifying T2DM patients with prevalent macrovascular complications. External validation in independent prospective multicenter cohorts and mechanistic studies are mandatory before clinical translation.

## Data Availability

The mass spectrometry proteomics data have been deposited to the ProteomeXchange Consortium via the iProx partner repository with the dataset identifier PXD085634.

## Supplementary material

Supplementary Figure 1 **(A)** Calibration plot of the combined model (clinical variables + four proteins) for discriminating diabetic macrovascular complications. The dashed red diagonal line represents perfect calibration. The blue curve shows the Loess-smoothed calibration curve with 95% confidence intervals. Quantitative calibration metrics were derived from logistic recalibration. The combined model demonstrated good calibration, with a calibration slope of approximately 1.000 and a Brier score of 0.032. **(B)** Decision curve analysis comparing the clinical model (age + disease duration + HbA1c) and the combined model (clinical variables + four proteins). The y-axis represents net benefit, and the x-axis represents threshold probability. The combined model provided greater net benefit than the clinical model across threshold probabilities of 0–50%, with the largest difference at intermediate thresholds (20–40%). The “Treat All” and “Treat None” curves are shown as references.

Supplementary Table 2 Full model, multicollinearity assessment, and correlation matrix.

Supplementary Table 3 FDR-adjusted correlation results.
